# Supplementary material for: Do soil health indicators predict carbon and nitrogen functional stability under drought and heat?
Source: PLoS One. 2025 Jun 6;20(6):e0325128. doi: 10.1371/journal.pone.0325128 (PMC12143542; doi:10.1371/journal.pone.0325128)
Supplement: S1 File — (DOCX) [file pone.0325128.s001.docx]

**Do soil health indicators predict carbon and nitrogen functional stability under drought and heat?**

**Supplementary Information**

1. Supplementary methods

Ten cores from each site were randomly allocated for baseline analyses. Bulk density was calculated using the core volume and oven-dry soil mass. Water-filled pore space was derived from the moisture and bulk density, assuming a particle density of 2.65 g cm^-3^ (Linn and Doran, 1984). Thermal properties were measured at field capacity and at field moisture, following the method of Haruna et al. (2017). To measure thermal properties at field capacity, for five cores from each site both ends were secured with cheesecloth and cores were placed vertically in a tub and gently saturated by slowly increasing the water level in the tub with tap water. After 48 h saturation, cores were removed from water and let to equilibrate at 21°C in a plastic bin lined with moist paper towels to maintain humidity. Conductivity, diffusivity, and resistivity were measured after 30 min and 1 h of equilibration using a KD2 dual-probe heat pulse sensor (Decagon Devices, Pullman, Washington). Two measurements were made per core, and the average was used. To determine thermal properties at field moisture, the same measurements were made on the second set of five cores, without saturation. This set was reserved for further analyses as described below.

Five baseline cores were sieved to <4 mm. A subsample was stored at -20°C for enzyme analysis, as described below. Gravimetric water content (GWC) was measured by drying field-moist soil for 24 hr at 105° C. Microbial biomass C and N were measured by chloroform fumigation extraction (Horwath and Paul, 1996). Briefly, one 5-g sample of field moist soil was extracted immediately with 25 ml 0.5 M K_2_SO_4_, while a second was fumigated with chloroform for 24 h, and then similarly extracted. Extracts from both were analyzed for extractable organic C (EOC) using a TOC Analyzer (Elementar, Germany). On the same extracts, total extractable N was measured using alkali persulfate digestion (Cabrera and Beare,1993). Microbial biomass C and N were calculated as the difference between C and N in fumigated and unfumigated soil extracts. No correction factor was used. Nonfumigated soil extracts were analyzed for ammonium (NH_4_-N) and nitrate (NO_3_-N) using the salicylate method (Verdouw et al., 1978; Forster, 1995) and a single reagent method (Doane and Horwath, 2003), respectively. Mineral N was calculated as the sum of NH_4_-N and NO_3_-N. Four additional subsamples were weighed into 40 ml glass vials. Two were mixed with ground residues from a multispecies cover crop mix (C:N =14:1) at a rate of 2.6 mg g^-1^ dry soil, corresponding to 1000 mg C kg^-1^ dry soil and 70 mg N kg^-1^ dry soil. Residue was mixed thoroughly with the soil, after which vials were adjusted to 60% water holding capacity (WHC) with deionized water and placed together uncovered into a 907-ml glass jar with an airtight lid fitted with a rubber septum for headspace sampling. The other two were treated similarly, except that no residue was added. Jars were incubated at 21 °C for 7 d. Jar headspace CO_2_-C was measured after 1 d, 3 d and 7 d on a gas chromatograph (Shimadzu, Japan). Jars were aerated after each sampling event. Each pair of vials was analyzed for MBC, MBN, and mineral N as described above. Potential C mineralization from soil organic matter was measured as the cumulative C evolved from the jar containing unamended vials. Apparent C and net N mineralization in response to residue addition was calculated as the difference in cumulative C respiration or mineral N between vials incubated with and without residue additions, while apparent MBC and MBN change in response to residue additions were calculated as the MBC and MBN difference between vials incubated with and without residue additions.

The remainder of the sieved soils were air-dried in a thin layer. Water-holding capacity (WHC) was determined gravimetrically. Texture was measured using the hydrometer method on a fraction sieved to <2 mm, from which organic matter had been removed by treating with H_2_O_2_ in a 90 °C water bath (Gee and Bauder, 1996). An index of wet aggregate stability was measured on 4 g of air-dry 1-2 mm aggregates using a Eijkelkamp wet sieving apparatus equipped with 53 µm sieve cups (Kemper and Roseneau, 1986). Total organic C and N were measured by dry combustion, after a dilute HCl test indicated that no carbonates were present (Nelson and Summers, 1996). Available P and base cations were assessed with a Mehlich-1 extraction (Mehlich, 1953) followed by analysis using inductively coupled plasma optical emission spectroscopy (ICP-OES) at the University of Georgia Soil, Plant, and Water Laboratory (Athens, Georgia, USA). Soil pH measured in a 2:1 water: soil slurry (Thomas, 1996). Permanganate-oxidizable C (POXC) was analyzed on duplicate 2.5 g samples of air-dry soil sieved to <2 mm (Culman et al., 2022). Short term C mineralization was measured as described by Lazicki et al. (2021), by rewetting 12 g samples of air-dried soil, sieved to <2 mm and placed in 50-ml glass beakers, to 60% WHC and incubating for 3 d at 25 °C in 1-L glass jars fitted with rubber septa for headspace sampling. For forest oils, 60% WFPS (using the volume 12 g unpacked soil in the beaker) was used instead as it was found that they required more water to wet thoroughly. Headspace samples were taken after 24, 48, and 72 h and analyzed for CO_2_-C by gas chromatography. After each sampling, the jars were evacuated by removing the lids and vigorously fanning. The values were added together to obtain total short-term C mineralization. The CO_2_-C mineralized after 24 h was divided by the MBC measured from undisturbed cores to determine the metabolic quotient (qCO_2_).

*Enzyme analysis*

Five randomly selected cores from each site were sieved to <4 mm. One subsample was stored at -20 °C for measuring the potential activity of four hydrolytic enzymes (β-glucosidase (BG), leucine aminopeptidase (LAP), N-acetyl-β-glucosaminidase (NAG), and β-xylosidase (XYL) using the microplate fluorescence method (Bell et al., 2013). Briefly, 2.75 g of soil were weight into a 15-ml microcentrifuge tube and stored at -20 C. Enzyme-specific fluorescent substrates were prepared according to Table 1.

| Enzyme abbreviation | Substrate | mg/100 mL DI H_2_O |
| --- | --- | --- |
| XYL | 4-MUB-β-D- xylopyranoside | 6.17 |
| BG | 4-MUB-β-D-glucopyranoside | 6.77 |
| NAG | 4-MUB-N-acetyl-β-D-glucosaminide | 7.59 |
| LAP | L-leucine-MUC hydrochloride | 6.5 |

Standard curves from 2.5 μM to 100 μM were prepared of 4-methylumbelliferone (MUB) for XYL, BG, and NAG and 7-amino-4-methylcoumarin (MUC) for LAP, using laboratory stock solutions. A buffer solution of 50 mM sodium acetate was prepared, and the pH adjusted to match average soil pH.

Thawed 2.75 g samples were added to a blender, along with 91 mL of buffer and blended on high for 1 minute. Contents were poured into a glass bowl, where they were lightly agitated using a stir bar. A multichannel pipette was used to transfer 800 ml of soil slurry into wells of three separate microplates, to which were added either MUC or MUB standard solutions, or the appropriate substrate as noted in Table 1. Plates were covered and mixed manually by inversion, and incubated at 25 °C for 4 hr, after which plates were centrifuged for 3 min at 1500 rpm, and 250 μL of supernatant were transferred to a flat-bottomed black 96-well plate. Fluorescence was measured on a microplate reader using an excitation wavelength of 365 and an emission wavelength of 450.

*Microbial community analysis*

Soil microbial DNA was extracted from five 0.25-g replicate subsamples of the frozen composite sample collected at each site using the Qiagen DNeasy PowerSoil kit, according to the manufacturer's instructions (Qiagen, Valencia, CA, United States). The extracted DNA was then sent to the University of Tennessee Genomics Core Facility where amplicon sequencing was conducted using paired-end sequences on the Illumina Miseq platform. Primer sets for 16S rDNA sequencing and ITS sequencing to were chosen according to Klindworth et al. (2014) and Cregger et al., (2018), respectively.

Demultiplexed sequences with the primers removed were run through the DADA2 pipeline using R (version 4.2.1) and low-quality regions and chimeric sequences were removed using the DADA2 pipeline (Callahan et al., 2017). Feature tables (ASVs) and feature data (representative sequences) were generated as resulting data from DADA2 pipeline. Feature data was compared against the SILVA and UNITE databases for bacterial and fungal classification, respectively, using the RDP Classifier with a threshold of 95% (Wang et al., 2007). ASVs with less than ten reads were removed. The ASV abundance tables were rarefied to a sequencing depth of 15,210 sequences for bacteria and 6,664 sequences for fungi, prior to calculating alpha diversity metrics.

**References**

Bell, C.W., Fricks, B.E., Rocca, J.D., Steinweg, J.M., McMahon, S.K., Wallenstein, M.D., 2013. High-throughput fluorometric measurement of potential soil extracellular enzyme activities. JoVE e50961. https://doi.org/10.3791/50961

Cabrera, M.L., Beare, M.H., 1993. Alkaline persulfate oxidation for determining total nitrogen in microbial biomass extracts. Soil Sci. Soc. Am. J. 57, 1007–1012. <https://doi.org/10.2136/sssaj1993.03615995005700040021x>

Cambardella, C.A., Elliott, E.T., 1992. Particulate soil organic matter changes across a grassland cultivation sequence. Soil Sci. Soc. Am. J. 56: 777-783

Cregger, M.A., Veach, A.M., Yang, Z.K., Crouch, M.J., Vilgalys, R., Tuskan, G.A., Schadt, C.W., 2018. The Populus holobiont: dissecting the effects of plant niches and genotype on the microbiome. Microbiome 6, 31. https://doi.org/10.1186/s40168-018-0413-8

Culman, S.W., Hurisso, T.T., Wade, J., 2021. Permanganate Oxidizable Carbon, in: Soil Health Series. John Wiley & Sons, Ltd, pp. 152–175. https://doi.org/10.1002/9780891189831.ch9

Callahan BJ, McMurdie PJ, Rosen MJ, Han AW, Johnson AJA, Holmes SP. 2016.
DADA2: High-resolution sample inference from Illumina amplicon data. 7. Nat Methods
13:581–583.

Doane, T.A., Horwáth, W.R., 2003. Spectrophotometric Determination of Nitrate with a Single Reagent. Anal. Lett. 36, 2713–2722. <https://doi.org/10.1081/AL-120024647>

Forster, JC. 1995. Soil nitrogen, in: Alef, K., Nannipieri, P., (Eds.), Methods in Applied Soil Microbiology and Biochemistry. Academic Press, San Diego, pp. 79-87.

Gee, G.W., Bauder. J.W., 1996. Particle-size analysis, in: Klute, A., (Ed.), Methods of Soil Analysis: Physical and Mineralogical Methods. Part 1. ASA and SSSA, Madison WI, pp. 383-411.

Haruna, S.I., Anderson, S.H., Nkongolo, N.V., Reinbott, T., Zaibon, S., 2017. Soil thermal properties influenced by perennial biofuel and cover crop management. Soil Sci. Soc. Am. J. 81, 1147–1156. <https://doi.org/10.2136/sssaj2016.10.0345>

Horwath, W.R., Paul, E.A., 1994. Microbial biomass, in: Weaver, R.W, Angle, S., Bottomley, P., Bezdiecek, D., (Eds.), Methods of Soil Analysis. Part 2. Microbiological and Biochemical Properties, 2^nd^ ed. ASA and SSSA, Wisconsin, pp. 753-773.

Jones, D.L., Willett, V.B., 2006. Experimental evaluation of methods to quantify dissolved organic nitrogen DON and dissolved organic carbon DOC in soil. Soil Biol. Biochem., 38, 991–999.

Kemper, W.D., Rosenau, R.C., 1986. Aggregate Stability and Size Distribution, in: Klute, A. 635 (Ed.), Methods of Soil Analysis, Part 1, Physical and Mineralogical Methods, Agronomy 636 Monograph. ASA and SSSA, Madison, WI, USA, pp. 425–442.

Klindworth, A., Pruesse, E., Schweer, T., Peplies, J., Quast, C., Horn, M., Glöckner, F.O., 2013. Evaluation of general 16S ribosomal RNA gene PCR primers for classical and next-generation sequencing-based diversity studies. Nucleic Acids Research 41, e1. https://doi.org/10.1093/nar/gks808

Lazicki, P., Geisseler, D., 2021. Relating indicators to soil health functions in conventional and organic Mediterranean cropping systems. Soil Sci. Soc. Am. J. 85, 1843–1857. https://doi.org/10.1002/saj2.20279

Linn, D.M., Doran, J.W., 1984. Effect of Water-Filled Pore Space on Carbon Dioxide and Nitrous Oxide Production in Tilled and Nontilled Soils. Soil Sci. Soc. Am. J. 48, 1267–1272. <https://doi.org/10.2136/sssaj1984.03615995004800060013x>

Nelson, D.W., Sommers, L.E., 1996. Total carbon, organic carbon, and organic matter, in: Sparks, D.L. (Ed.), Methods of Soil Analysis. Part 3, Chemical Methods, second ed. ASA and SSSA, Wisconsin, pp 961-1010

Thomas, G.W., 1996. Soil pH and soil acidity, in: Sparks, D.L. (Ed.), Methods of Soil Analysis. Part 3, Chemical Methods, second ed. ASA and SSSA, Wisconsin, pp. 475-490.

Wang, Q., Garrity, G.M., Tiedje, J.M., Cole, J.R., 2007. Naïve Bayesian Classifier for Rapid Assignment of rRNA Sequences into the New Bacterial Taxonomy. Appl Environ Microbiol 73, 5261–5267. https://doi.org/10.1128/AEM.00062-07
