# Supplementary figures and images for: Do soil health indicators predict carbon and nitrogen functional stability under drought and heat?
Source: PLoS One. 2025 Jun 6;20(6):e0325128. doi: 10.1371/journal.pone.0325128 (PMC12143542; doi:10.1371/journal.pone.0325128)

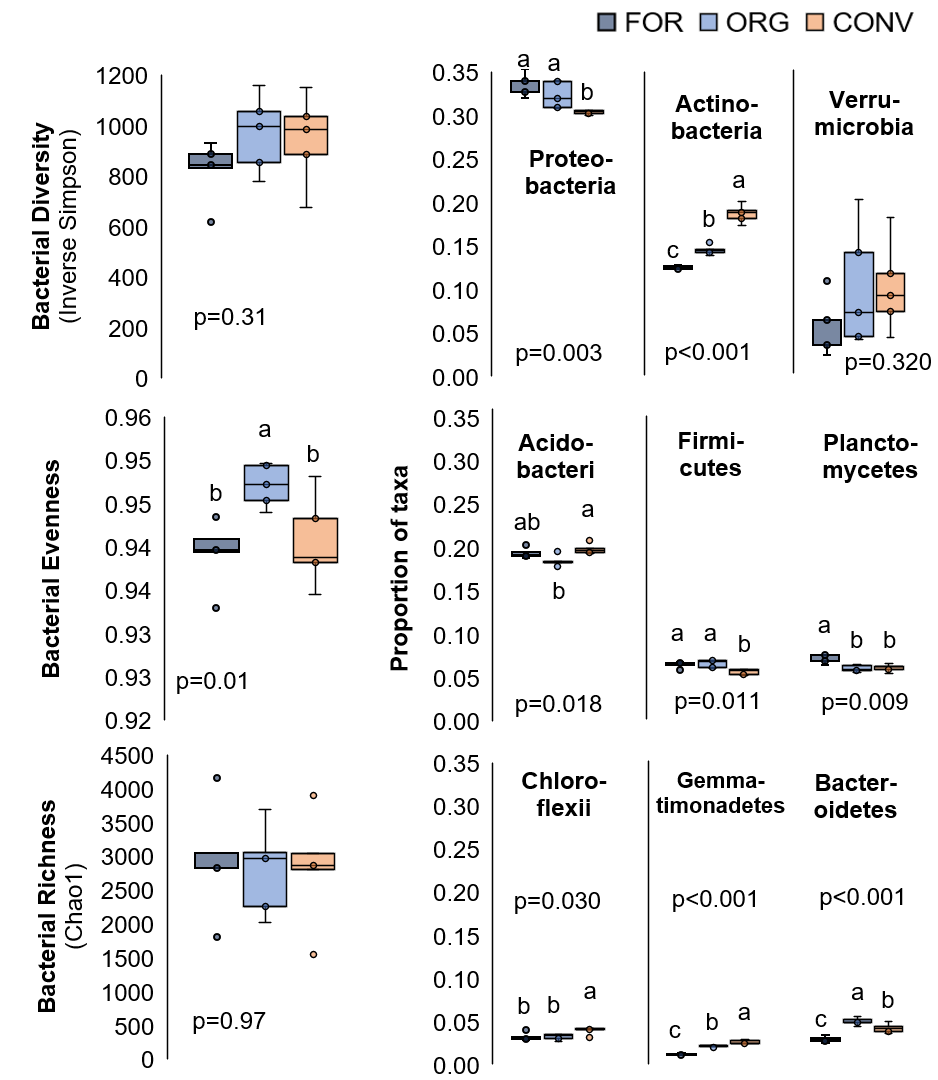

Supplement: S1 Figa — FOR= Forest soil ORG = soil from a long-term organic annual cropping system CONV = soil from a long-term conventionally-managed cropping system. Different lowercase letters represent statistical difference at p < 0.05 using Tukey HSD (n = 5). (TIF) [file pone.0325128.s002.tif]

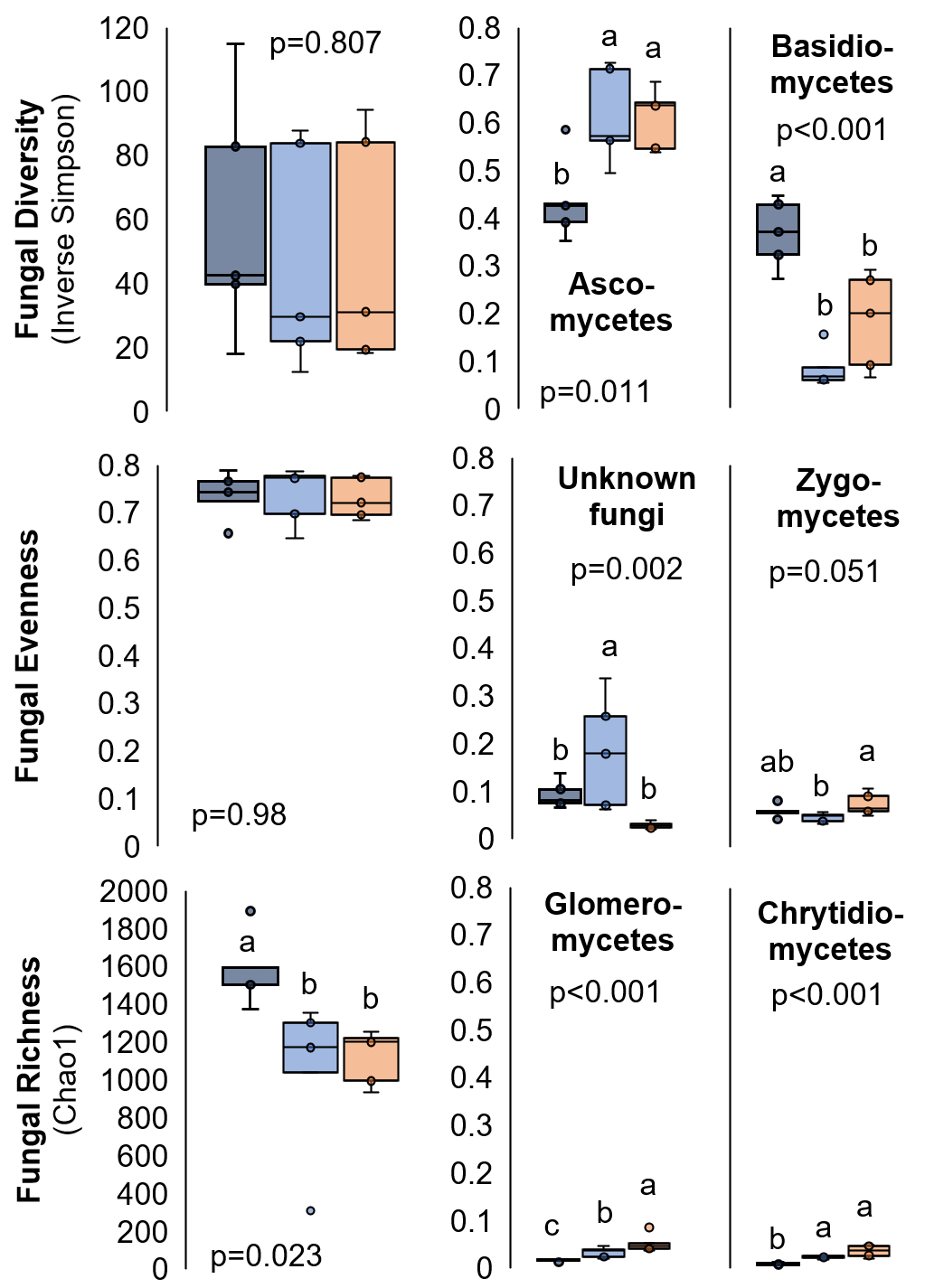

Supplement: S1 Figb — FOR= Forest soil ORG = soil from a long-term organic annual cropping system CONV = soil from a long-term conventionally-managed cropping system. Different lowercase letters represent statistical difference at p < 0.05 using Tukey HSD (n = 5). (TIF) [file pone.0325128.s003.tif]
