# Supplementary material for: Do soil health indicators predict carbon and nitrogen functional stability under drought and heat?
Source: PLoS One. 2025 Jun 6;20(6):e0325128. doi: 10.1371/journal.pone.0325128 (PMC12143542; doi:10.1371/journal.pone.0325128)
Supplement: Attachment — Submitted filename: Response to Reviewers_R1.docx [file pone.0325128.s007.docx]

Response to Reviewers: PONE-D-24-46379

We thank the reviewers very much for taking the time to provide these thoughtful reviews. Their suggestions have greatly improved the paper; we address them individually below. Please note that the line numbers refer to the version of the manuscript with all changes tracked and visible.

**Response to the editor**

1. REMOVE FUNDING INFO FROM ACKNOWLEDGEMENTS

The info has been removed. The acknowledgements now read (Lines 474-476):

*“Thanks to Nathan Hicklin for assistance in site identification and sampling, and to Windy Acres and TC Groves Farms for allowing us to sample their soils. Thanks to Maddie Evans, Michael Evan Russell and Mary Catherine Pile for help in sample analysis.”*

2. In your Methods section, please provide additional information regarding the permits you obtained for the work. Please ensure you have included the full name of the authority that approved the field site access and, if no permits were required, a brief statement explaining why.

We received verbal permission to sample all sites from the growers. We have added a sentence to the methods section (Lines 108-109)

*“Sites were accessed with grower permission.”*

3. We noted in your submission details that a portion of your manuscript may have been presented or published elsewhere. Please clarify whether this [conference proceeding or publication] was peer-reviewed and formally published. If this work was previously peer-reviewed and published, in the cover letter please provide the reason that this work does not constitute dual publication and should be included in the current manuscript.

The incubation portion of the experiment was published in Applied Soil Ecology (<https://doi.org/10.1016/j.apsoil.2023.104947>). In that paper, we report selected C and N dynamics of our three different soils under drought or drought+heat stress. In the paper we have submitted for consideration in PLOS one, we use these values to calculate multifunctional resistance and resilience indices, and report the relationship of these indices to a suite of soil health and microbial assays. Thus, all of the data presented in the two papers is different, as are the experimental questions asked. We felt

4. In the online submission form, you indicated that your data will be submitted to a repository upon acceptance.  We strongly recommend all authors deposit their data before acceptance, as the process can be lengthy and hold up publication timelines. Please note that, though access restrictions are acceptable now, your entire minimal  dataset will need to be made freely accessible if your manuscript is accepted for publication. This policy applies to all data except where public deposition would breach compliance with the protocol approved by your research ethics board. If you are unable to adhere to our open data policy, please kindly revise your statement to explain your reasoning and we will seek the editor's input on an exemption.

We have submitted the data to Dryad but it has not yet been peer reviewed. The reviewer link can be found here: [http://datadryad.org/share/5YfR68Tg6rR8EVE3RyLQ9s3qj7PwDFnNVarkANEWYCA](http://h52wvrds.r.us-west-2.awstrack.me/L0/http:%2F%2Fdatadryad.org%2Fshare%2F5YfR68Tg6rR8EVE3RyLQ9s3qj7PwDFnNVarkANEWYCA/1/01010195ef45bd13-549a4959-d0e5-462e-a67c-99dbc6b8e44b-000000/47chYTWTPimw08_BRFg7CvPU4pg=420).; the permanent link (currently private) is here: <https://doi.org/10.5061/dryad.1c59zw45q>

5. PLOS requires an ORCID iD for the corresponding author in Editorial Manager on papers submitted after December 6th, 2016. Please ensure that you have an ORCID iD and that it is validated in Editorial Manager. To do this, go to ‘Update my Information’ (in the upper left-hand corner of the main menu), and click on the Fetch/Validate link next to the ORCID field. This will take you to the ORCID site and allow you to create a new iD or authenticate a pre-existing iD in Editorial Manager.

Dr. Lazicki’s ORCID ID has been validated in Editorial Manager: <https://orcid.org/0000-0002-2481-3116>

6. Thank you for stating the following in the Acknowledgments Section of your manuscript:

“USDA ARS (grant number 58-6066-8-043); Tennessee Department of Agriculture (grant number A20-0337)”

We note that you have provided additional information within the Acknowledgements Section that is not currently declared in your Funding Statement. Please note that funding information should not appear in the Acknowledgments section or other areas of your manuscript. We will only publish funding information present in the Funding Statement section of the online submission form.

Please remove any funding-related text from the manuscript and let us know how you would like to update your Funding Statement. Currently, your Funding Statement reads as follows:

“USDA ARS (grant number 58-6066-8-043); Tennessee Department of Agriculture (grant number A20-0337)”

Please include your amended statements within your cover letter; we will change the online submission form on your behalf.

Thank you! We have removed the information from the Acknowledgments. The current funding statement (“USDA ARS (grant number 58-6066-8-043); Tennessee Department of Agriculture (grant number A20-0337)” is correct.

**Response to reviewers**

Reviewer #1: The manuscript titled "Do soil health indicators predict carbon and nitrogen functional stability under drought and heat?" delves into a critical and contemporary issue: the intricate relationship between soil health indicators and the functional stability of soil carbon (C) and nitrogen (N) cycling in the face of climate-induced stress. Unraveling how soil health modulates resistance and resilience to drought and heat is paramount for sustainable soil management and effective climate change adaptation. This study offers profound insights into the differential impacts of various management practices—forest, conventional, and organic—on soil functional stability. The strategic selection of three distinct land-use types serves as an ideal framework for comparing soil health and functional stability across diverse agricultural approaches. The sampling methodology, which encompasses the collection of undisturbed soil cores and bulk samples, is meticulously crafted to encapsulate both spatial and functional variability.

The experimental setup, encompassing both drought and combined drought-heat stress scenarios, is pertinent to projected climate change models. The 28-day stress and recovery period is adequately designed to capture significant alterations in soil functions. Utilizing a multifunctional approach to evaluate carbon and nitrogen cycling provides a robust assessment of soil functional stability. Employing 16S and ITS sequencing for analyzing microbial community diversity and composition offers a rigorous methodology to elucidate the role of microbial communities in maintaining soil function. The comprehensive inclusion of both bacterial and fungal communities aligns with contemporary understanding in soil microbiology.
The findings that microbial community composition exhibits significant variation among different sites, while diversity metrics remain relatively consistent, suggest that specific taxa or community structure may play a more crucial role than overall diversity in determining functional stability. Identifying key soil properties such as pH levels, chemical fertility, and porosity that differentiate these sites is essential for elucidating the underlying mechanisms governing soil functional stability.

The results indicate that resistance (RS) and resilience (RL) exhibit variability depending on the type of stressor and land use practices, underscoring the intricate nature of soil functional stability. Notably, the findings reveal that the addition of heat to drought conditions significantly diminishes RS and RL, a critical insight for regions subjected to multiple climate stressors. Furthermore, the observation that organic management practices generally promote higher RL compared to conventional and forested sites implies that such practices may play a pivotal role in enhancing soil functional stability.

The application of principal components analysis (PCA) and stepwise multiple regression to identify predictors of RS and RL is statistically robust. The discovery that soil health indicators, such as total organic carbon and microbial biomass, are correlated with nitrogen cycle resilience under drought stress, is crucial for the development of comprehensive soil health assessment frameworks. The absence of a consistent relationship between microbial diversity and functional stability implies that other factors, including community composition and soil properties, may exert a more substantial influence.

The study concludes that conventional soil health indicators, as traditionally defined within agricultural contexts, may not comprehensively capture the functional stability of natural ecosystems. The organic management system appears to effectively balance the advantages of high soil organic matter and microbial activity with sufficient chemical fertility, thereby enhancing functional stability. This conclusion is robustly supported by empirical data and offers practical guidance for sustainable soil management practices.

1. In Section 2.4.3, the equations for calculating RS and RL are clearly presented; however, incorporating additional explanatory text would enhance readers' comprehension of the rationale underpinning these calculations.

This is an excellent point. We have added some text to the paragraph in question, explaining some of the rationale. The introduction to the equations now reads (lines 203-207):

*“Resistance (RS) and resilience (RL) indices were calculated for each function using the method of Orwin and Wardle [22]. This method is commonly used in microbial response studies as it, a) increases monotonically as RS or RL increase, b) gives equivalent values for a response of the same magnitude, whether it is higher or lower than that of the control, c) avoids infinite values and zero denominators and d) standardizes the recovery value to the initial disturbance [22].”*

2. Several sections include redundant information. For instance, the analysis of microbial community diversity and its correlation with functional stability is addressed repeatedly across multiple sections. To enhance clarity and conciseness, this information should be consolidated.

We have gone through the MS and looked for redundant statements so that they can be consolidated.

3. Some sentences are complex and could be simplified for clarity. For example, in Section 3.3, the sentence "This suggests that in the unfertilized, acidic FOR soil, soil chemistry posed a stronger control on community RL to DRT than factors traditionally considered in a soil health assessment" This sentence could be divided into two sentences for improved readability.
The manuscript is scientifically rigorous and offers valuable insights into the relationship between soil health indicators and functional stability under climate stress. The experimental design is robust, and the results are thoroughly supported by comprehensive data. While the manuscript is well-written, there are minor grammatical and stylistic enhancements that could further improve clarity. This study makes a significant contribution to the fields of soil science and sustainable agriculture.

We have gone through the MS and looked for places where sentences can be simplified without losing their logical flow, and where grammar can be improved. Examples include Lines 125-128, Lines 141-144, Lines 162-164, Lines 172-174, Lines 182-186

Reviewer #2: This study provides valuable insights into the complex relationships between soil health indicators and functional resilience under climate stress. By linking carbon and nitrogen cycling responses to different management practices, it highlights key factors influencing soil resistance and resilience. The findings offer a strong scientific basis for improving soil management strategies under climate change. However few points are to be properly addressed for the clarity of the readers (appended below):

1. The introduction mentions microbiota but could briefly specify key microbial groups (bacteria, fungi, archaea) that drive carbon and nitrogen cycling.

Addressed in line 45-46. The sentence now reads:

*“These extreme climate events can all be stressors on the bacteria, fungi, and archaea that drive important soil functions such as carbon (C) and nitrogen (N) cycling [1,2].”*

2. The explanation of RS and RL should be refined for clarity. Instead of "more quickly and completely to regain function," use "recover function more rapidly and effectively."

Addressed in line 50-53 with a slight change; it’s important to note that resemblance to non-stressed conditions is an important component of RS, and this change is defined both in terms of how long it takes, and how far the stressed and non-stressed values are from each other.:

*“**That is, important microbially-driven functions in “healthy” soils are assumed to change less during stress (RS) and to more rapidly and closely resemble those of non-stressed soils when conditions return to normal (RL) [3].”*

3. While the introduction discusses soil organic carbon (SOC), porosity, and fertility, it should explicitly state which soil health assessment methods are commonly used and how they may overlook stress response.

We have added a sentence to clarify broadly the types of soil health metrics commonly used (e.g. various pools and processes—there are a lot of soil health assessment schemes, and it would take too long to list them all), and that these schemes don’t measure how these pools or processes respond to climate stress. (Lines 55-58)

*“However, soil health assessment schemes, which normally include soil organic matter and its various fractions, available nutrients, physical properties, and indices of soil biological activity such as respiration or potential enzyme activities, generally do not include how these pools and functions respond to climate stress (e.g. [6,7]).”*

4. The transition to multi-functionality indices should be smoother, explaining their relevance in quantifying overall ecosystem function rather than listing citations.

Excellent point! We have added some more explanation on the benefits of multifunctionality indices (Lines 77—81:

*“This approach has long been used in ecology to study the links between organisms and ecosystem function, as it provides a means of summarizing the aggregate functional change of an ecosystem in response to a change that simultaneously affects all members of the community [16]. More recently, the approach has been combined with RS and RL indices to relate the stress behavior of soil processes to soil and microbial community characteristics [17,18].”*

5. The hypotheses should be more precise:
- Specify how high porosity and SOC reduce water loss and heat transmission.
- Explain why microbial diversity may enhance both RS and RL by fostering functional redundancy.

We have made the suggested changes. The hypotheses now read (Lines 90-96):

*“1) Adding heat to drought would decrease RS and RL of all measured C and N cycle metrics at all sites [11], 2) RS would increase with soil porosity and soil C, as such soils will likely lose water and transmit heat more slowly due to better water holding capacity and improved thermal resistivity [10,19], 3) High chemical fertility would be associated with high RL, as more available nutrients will support faster microbial regrowth [8], and 4) Higher bacterial and fungal diversity would be associated with high RS and RL, as increased functional redundancy increases the likelihood of organism’s ability to perform any given function under both stress and recovery conditions [18].”*

6. The methodology section should be more structured by separating different processes with clear subheadings. Consider breaking down complex sentences for readability.

We carefully reread the methods and broke up some of the longer sentences. Examples include Lines 125-128, Lines 141-144, Lines 162-164, Lines 172-174, Lines 182-186

If we understand the comment correctly, the reviewer suggests grouping C and N cycling processes separately. While this suggestion is reasonable, the nature of the experiment is to measure the change in many processes simultaneously in response to an imposed stress and recovery. Therefore, we think that it would be more difficult to describe the experiment if we structured by separate processes rather than by chronology of laboratory steps (e.g. baseline soil properties, imposition and lifting of stress, and various functional measurements performed at each timepoint), as it is currently organized. Additionally, separating C and N cycling functions would greatly lengthen the manuscript as it would involve describing or at least referencing the same experiment twice where both C and N cycling functions were measured. We think this would also reduce clarity as it might be less evident that many functions (e.g. C and N mineralization, MBC, MBN, C- and N-cycling activities) were all measured on the same physical soil core in the course of the same experiment.

To improve the clarity of which functions were measured when, we have re-ordered so the paragraphs describing which functions were designated as either C or N -cycling are located directly after the description of the incubation experiment, under the subhead "C and N cycling functions”. We also added some language to make it clearer on which fractions the cores were measured. Unfortunately, due to journal style rules, we could not add an additional subhead for each paragraph, but we added language to make it immediately clear whether it referred to C or N cycling. We hope that these changes address the reviewer’s concerns. The paragraphs read (lines 187-200)

*“Functions were designated as related to C or N cycling. The nine N cycling functions were a) From the undisturbed cores: mineral N, the proportion of the mineral N measured which was in the form of NH4, MBN, EON, and the potential activity of the enzymes leucine aminopeptidase (LAP) and N-acetyl-β-glucosaminidase (NAG) from undisturbed cores; b) From unamended, sieved soil during incubation: net N mineralized over 7 d and c) From residue-amended soil after 7 d incubation: additional mineral N and MBN measured, compared to unamended soils.*

*The ten C cycling functions were: a) From the undisturbed cores: cumulative total respiration per unit MBC, MBC, EOC, and the potential activity of the enzymes β-glucosidase (BG) and xylanase (XYL) b) From unamended soil during incubation: CO2-C mineralized over 24 h and 7 d, CO2-C mineralized over 24 h per unit MBC (qCO2), and c) From residue-amended soil over 7 d incubation: additional MBC and CO2-C measured, compared to unamended soils.”*

7. Ensure uniformity in naming conventions, such as consistently referring to "CONV," "ORG," and "FOR" throughout the text without alternation between abbreviations and full forms.

We have done a text search throughout the MS and eliminated any alternation. However, we have kept the unabbreviated form when used in reference to something other than our experimental soil descriptors (e.g. organic matter rather than ORG matter). We have similarly corrected some inconsistencies in SOM/SOC.

8. In certain methods, like enzyme activity assays and microbial community analysis, Briefly summarizing key steps within the main text will enhance clarity.

Because of the very large number of analyses, and because we followed standard methods, we elected to describe our sampling and storage procedures but referred the reader to the standard method for more analytical details. Otherwise, the methods section would be prohibitively long. We have included a detailed method for DNA extraction, community analysis, and enzyme analysis in the Supplemental methods. If the reviewer strongly feels that there are more details that it’s important to include in the main text we can consider adding these. At the moment, we added some wording to briefly orient the reader to the specific method used (lines 128-133)

*“Subsamples were stored at -80°C for microbial community quantification via 16s and ITS analyses and at -20 °C for enzyme analysis. Soil microbial DNA was extracted from five 0.25-g replicate subsamples of the frozen composite sample collected at each site using the Qiagen DNeasy PowerSoil kit, according to the manufacturer's instructions (Qiagen, Valencia, CA, United States). Enzyme analyses were performed using the fluorescence microplate method (Table 1). Details of DNA extraction, community analysis, and enzyme assays are given in the Supplemental Methods.”*

9. Ensure uniformity in how measurements are reported (e.g., using consistent units for soil moisture and enzyme activities). Some values, such as temperatures and durations, should be explicitly stated in standard scientific formats.

Temperatures are stated in °C , durations as h or d when used as a unit, or “day” if referring to a particular sampling day, e.g. ”on day 28”. Moisture content has been changed from “g H_2_O g^-1^ soil” to “a water content of X g g^-1^ dry soil”. Enzyme measurements are not reported. A text search was performed to ensure consistency in measurements.

10. How do microbial community composition and diversity vary across forest, organic and conventional land-use systems?

We have included this information in the paragraph under subhead 3.1.1 To make this clearer, we have altered the heading to read “*Variations in microbial communities across land-use types”* The paragraph reads (Lines 261-275):

*“For both bacteria and fungi, community composition varied more than alpha diversity. PERMANOVA analysis showed that bacterial communities differed significantly among sites (p=0.002). Major phyla whose relative abundance differed significantly among sites were Planctomycetes (FOR>CONV=ORG; p=0.009), Proteobacteria (FOR=ORG>CONV, p=0.003), Firmicutes (ORG=FOR>CONV; p=0.01), Bacteroidetes (ORG>CONV>FOR; p<0.001), Actinobacteria and Gemmatimonadetes (CONV>ORG>FOR; p<0.001 for both), Acidobacteria (CONV>ORG; FOR intermediate, p=0.02), and Chloroflexii (CONV>ORG=FOR) (Supplemental information, Figure S1A. The only major phylum with similar abundance in all three sites was Verrumicrobia. Bacterial evenness was significantly higher in the ORG site than in the other two (F=7.4, p=0.01). However, bacterial richness and diversity, as measured by the Chao 1 and Inverse Simpson indices, respectively, did not differ among sites. Fungal richness was significantly higher in the FOR site than the other two (p=0.002), while diversity and evenness were similar (Supplementary Figure S1B). Fungal communities, like bacterial, differed significantly by site (p=0.002). Major fungal phyla which differed significantly among sites were Basidiomycetes (FOR>CONV=ORG; p<0.001), Ascomycetes (ORG=CONV>FOR, p=0.01), Chytridiomycetes (CONV=ORG>FOR, p<0.001), and Glomeromycetes (CONV>ORG>FOR, p<0.001).”*

11. What are the key soil physicochemical properties driving microbial community shifts under different land-use types.

We agree that this would be interesting to comment on. However, our experiment was not designed to test this question. Had we experimentally manipulated soil physicochemical properties or sampled along a gradient, we might have been able to give results about microbial community drivers. Since we are just reporting observations from three sites under very different long-term management, in order to avoid undue speculation we only report the observed differences in soil physicochemical properties and the attendant differences in community composition.

12. How do carbon and nitrogen cycling processes respond to single and combined drought-heat stress across distinct land-use systems.

The purpose of this paper is to document the association between measured soil health metrics and RS and RL indices. We have reported the effects of single and combined drought-heat stress across land use systems in a companion paper (Lazicki et al., 2023), to which the reader is referred in line 314-315

13. What mechanisms underpin microbial functional resilience and resistance to environmental stressors in different land-use regimes?

We have noted several potential mechanisms throughout the results/discussion. For example:

**FOR high RS to DRT but not D+H** (lines 339-348): “*A possible explanation is that soils with less history of drought stress have a larger response to drought than more adapted soils [27,28]. This provides evidence for the theory that stress response is affected by microbial community adaptation as well as to the magnitude of the stress and the soil environment [29]. This mechanism likely explains the high RS to DRT but low RS to D+H in the shaded, litter-covered FOR site. Similarly, Guillot et al. [11] measured the lowest microbial biomass RS to D+H in soils collected from under the tree row in an agroforestry experiment, while the highest RS to D+H was measured in soils collected from the cropped inter-row. Additionally, analysis of a network of climate change studies across the US found increased proteolytic activity in temperate forests under warming and drought [30]. Such increased activity, combined with a lower microbial biomass to immobilize the products, may also have contributed to the low FOR site RS to D+H.”*

**Relative high RS of forest sites, RL of arable sites** (lines 372-375) “*We predicted that RS would increase with soil porosity and SOM (hypothesis 2), and that soils with higher chemical fertility will tend to have higher RL (hypothesis 3). In partial support of these hypotheses, “Arableness” (Prin 1; high fertility, low porosity) had significant negative correlations with multifunctional C and N cycle RS to DRT (Table 2), reflecting the FOR site’s numerically higher values than the arable sites for both C and N cycle RS to DRT.*”

**FOR poor RL to all stresses** (lines 378-382):” *Interestingly “Soil health” (Prin 2, which loaded for properties related to microbial biomass, activity, and aggregate stability) was not a good predictor of stress RL (Table 2).This suggests that in the unfertilized, acidic FOR soil, soil chemistry posed a stronger control on community RL to DRT than factors traditionally considered in a soil health assessment, such as structure and SOM”*

**CONV soil poor N cycle RL to DRT, better RL to D+H** (lines 395-403): *“Conversely, “Soil Health” was a strong positive predictor of N cycle RL to DRT. This result is in line with the hypothesis that in general, ecosystems with a high degree of internal self-regulation will be more buffered to external disturbances [5,9]. Under this theory, cycles which rely more heavily on synthetic inputs, such as N, would have low RS and RL to a stress which affects the pool or process in question. In our experiment, the synthetically fertilized CONV site with its high residual NO_3_-N and low microbial biomass, microbial activity and SOM is an example of a system with little power of internal N cycling [35]. The hypothesis that N cycle RL in the CONV system was limited by its low SOM is supported by the higher RL observed under D+H in the CONV system, as the addition of heat stress increased dissolved organic C concentrations [20], which would alleviate this limitation.”*

14. How can land management practices be optimized to enhance microbial stability and ecosystem functions under climate variability?

We have rewritten the first part of the conclusions to directly address this question (lines 422-441:

*“4.1 Implications for optimizing land management practices for functional stability under climate variability*

*The goal of this study was to link soil health status with stress response in three differently managed systems. Our results demonstrate that the long-term practice of management designed to improve soil health appeared to buffer soil RS and RL to climate stress. The ORG soil, which combined long perennial phases with periodic additions of manure and pH adjustment, appears to represent a “middle path” between the conventionally farmed and natural sites. That is, it created an environment characterized by good physical characteristics and high SOM like the natural site, which were associated with greater RS. At the same time, it possessed the adequate chemical fertility and neutral pH of the conventionally farmed site. These resulted in an RS and RL which was always equal to or greater than that of the other two systems, under both stresses. Conversely, conventional practices, even when combined with no-till, led to a less buffered system with relatively low RS to DRT, and very low RL of N cycling processes to DRT. This low N-cycle RL was especially associated with low SOM, biological activity, and porosity, combined with high soil NO3-N and a microbial community which may be more adapted to low-OM conditions.*

*Despite their wide variation in soil health characteristics, all systems showed poor RS when subjected to heat under drought conditions. An implication is that management which buffers soil temperature, such as the presence of living cover, high-albedo mulch, or crop residues, is a vital component of maintaining functional stability in a warming climate. It is especially important for natural systems to avoid conditions that expose the soil to unaccustomed stresses, as sites with low chemical fertility and an acidic pH may not be able to recover function as quickly.*

15. How can soil health assessment frameworks be modified to better account for the unique resilienc”e and stress responses of natural ecosystems, rather than relying solely on agricultural benchmarks?

We have rewritten the second part of the conclusion to more specifically address soil health assessment.. Since our focus is on agricultural soil health assessment and this is the primary context under which soil health assessment is performed, we do not address soil health assessment for natural systems.(Lines 442-461)

*“4.2 Implications for soil health assessment*

*Our results broadly support the assumption that more healthy soils, by traditional agriculturally-focused definitions (e.g. higher SOM and its fractions, microbial activity, and aggregate stability), are more resistant and resilient than less healthy ones. However, our findings highlight the limitations of soil health assessment as applied to natural systems. The “soil health” concept did not fit the FOR soil, with its high porosity, SOM, and microbial biomass but poor aggregation, high acidity, low fertility, and low enzyme activity. This is reasonable, since soil health is normally assessed in the context of agriculture in which a primary functional goal is sustained crop yields. As noted by Peterson et al. [9], creating a resilient agroecosystem may first require adding the external inputs needed to get it to a productive state (e.g., lime, fertility sources), and then managing so as to maximize its ability to internally self-regulate (e.g. improving SOM, structure, and biological activity). Our natural site’s generally good physical characteristics were associated with high RS to DRT for both C and N cycles. However, it had very poor RS when heat was added, likely as a result of a non-adapted microbial community. In addition, its acidity and low chemical fertility were strongly associated with poor stress RL. Our results imply that if the idea of “soil health” for agricultural systems includes the ability to respond well under a stress to which agricultural systems are more subject (such as heat), soil health assessment schemes that propose using metrics from an undisturbed native soil as benchmarks (e.g. [45]) may not be appropriate.”*

16. What specific microbial adaptations contribute to improved resistance and resilience under heat stress, and how can these be leveraged to enhance soil health in both natural and managed systems?

This is a very interesting question, but unfortunately our results do not shed direct light on it. Under our experimental conditions, none of the systems showed RS to heat stress (though the FOR system had the most difference between heat and drought). We have added a short paragraph to emphasize the importance of creating environments that are physically buffered against heat stress, given the poor intrinsic RS of all three systems (lines 436-441):

*“Despite their wide variation in soil health characteristics, all systems showed poor RS when subjected to heat under drought conditions. An implication is that management which buffers soil temperature, such as the presence of living cover, high-albedo mulch, or crop residues, is a vital component of maintaining functional stability in a warming climate. It is especially important for natural systems to avoid conditions that expose the soil to unaccustomed stresses, as sites with low chemical fertility and an acidic pH may not be able to recover function as quickly.“*
